# Clinical Presentation of, Treatment Approaches for, and the Impact of Social Determinants of Health on Pediatric Catatonia: A Structured Qualitative Synthesis and Targeted Literature Review

**DOI:** 10.7759/cureus.116791

**Published:** 2026-09-23

**Authors:** Raquel Gonzalez, Savraj S Sekhon, Sarah Tedesco

**Affiliations:** 1 Clinical Medicine, American University of Antigua, St. John's, ATG; 2 Psychiatry and Behavioral Sciences, Richmond University Medical Center, Staten Island, USA; 3 Psychiatry, The Milton S. Hershey Medical Center, Hershey, USA; 4 Psychiatry, Richmond University Medical Center, Staten Island, USA

**Keywords:** electroconvulsive therapy (ect), excited catatonia, pediatric catatonia, social determinants of health (sdoh), subtypes of catatonia

## Abstract

Catatonia is a complex neuropsychiatric syndrome with diverse presentations, including stuporous, excited, malignant, and periodic forms. While well documented in adults, pediatric catatonia remains underrecognized, with limited literature addressing its presentation, treatment, and the influence of social determinants of health (SDOH). This structured qualitative synthesis and targeted literature review examine eight pediatric patients with catatonia to identify clinical patterns, contributing factors, treatment strategies, and the impact of SDOH on diagnosis and outcomes.

Clinical presentations ranged from mutism and waxy flexibility in stuporous catatonia to agitation and self-injurious behavior in excited forms. Neurodevelopmental and psychiatric conditions, including autism spectrum disorder (ASD) and psychotic disorders, were common predisposing factors. SDOH and other contextual factors, including trauma, mental-health stigma, barriers to specialized care, and socioeconomic hardship, were variably reported and, in several cases, were associated with delayed recognition or access to treatment.

Benzodiazepines, particularly lorazepam, were effective in symptom management across cases, while antipsychotic medications provided adjunctive benefit in patients with prominent psychotic features. Electroconvulsive therapy (ECT) was a life-saving intervention in refractory cases but remained limited by systemic and legislative barriers. Supportive measures, including hydration and enteral feeding, were critical in severe presentations. Long-term management required individualized pharmacologic strategies to reduce relapse risk. This synthesis highlights the importance of early recognition, trauma-informed care, and addressing systemic inequities to improve outcomes in pediatric catatonia.

## Introduction and background

Catatonia is a complex neuropsychiatric syndrome characterized by a range of motor, behavioral, and autonomic abnormalities, including stupor, immobility, agitation, mutism, and autonomic instability [1]. It is commonly classified into four broad subtypes: stuporous, excited, malignant, and periodic. Stuporous catatonia presents with psychomotor retardation and immobility, whereas excited catatonia is associated with prolonged agitation. Malignant catatonia is the most severe form and may involve autonomic instability, including abnormal blood pressure, heart rate, or temperature regulation, as well as life-threatening complications [1]. According to Diagnostic and Statistical Manual of Mental Disorders, Fifth Edition (DSM-5) criteria, catatonia is diagnosed by the presence of at least three characteristic psychomotor features, including stupor, mutism, negativism, posturing, agitation, or echolalia [2]. In clinical practice, assessment may be guided by DSM-5 criteria or by validated instruments such as the Bush-Francis Catatonia Rating Scale, a widely used 23-item tool for the identification and assessment of catatonic symptoms [3,4]. Pediatric patients with untreated malignant catatonia may deteriorate rapidly, and adolescents with catatonia occurring in the context of psychotic or mood disorders have been shown to carry a significantly increased risk of premature mortality [5].

Catatonia may arise from a wide range of psychiatric, neurologic, autoimmune, metabolic, and toxic etiologies, yet it is frequently misdiagnosed or overlooked [6]. This heterogeneity may contribute to diagnostic delay, as pediatric catatonia can be mistaken for primary neurologic disease, medication effects, behavioral disturbance, or worsening psychiatric illness. Although extensively studied in adults, pediatric catatonia remains relatively underrepresented in the literature. In one pediatric psychiatric outpatient sample, catatonia was identified in 5.5% of children and adolescents evaluated, underscoring the need for heightened clinical awareness and prompt intervention [7]. Delayed recognition and treatment in children and adolescents can result in severe morbidity and, in some cases, fatal outcomes [8].

Social determinants of health (SDOH), as defined by the Office of Disease Prevention and Health Promotion, encompass the conditions in which individuals are born, live, learn, work, play, worship, and age. These determinants are broadly categorized into five domains: economic stability, education access and quality, healthcare access and quality, neighborhood and built environment, and social and community context [9]. Disruptions within these domains can significantly impede access to timely psychiatric evaluation, diagnosis, and treatment, particularly in vulnerable pediatric populations. In pediatric populations, these factors may delay recognition of catatonia by limiting access to psychiatric care, prolonging untreated psychiatric illness, increasing caregiver burden, and contributing to stigma surrounding mental health treatment. 

Current treatment strategies for catatonia emphasize early identification and prompt initiation of benzodiazepines, typically beginning with a lorazepam challenge. Electroconvulsive therapy (ECT) remains a highly effective intervention for severe or treatment-resistant cases [10]. Atypical antipsychotics, including risperidone, have demonstrated benefit in select presentations, particularly when psychotic symptoms are prominent, though their precise role in catatonia remains incompletely understood [6,11]. 

Recent pediatric case reports describe catatonia across neurodevelopmental, psychotic, mood, and trauma-related presentations [12-19]. Across these reports, the availability and detail of SDOH information varied, with some describing contextual or structural barriers relevant to recognition or treatment.** **

To our knowledge, few pediatric catatonia reports have systematically examined the role of SDOH alongside clinical presentation and treatment response. This study combines a structured qualitative synthesis of eight previously published pediatric catatonia case reports with a targeted literature review. We further examine how SDOH, including socioeconomic status, family dynamics, cultural context, and access to care, were reported in relation to diagnostic delay, treatment course, and recovery. By identifying clinical and contextual patterns across reported cases, this study aims to inform more equitable, trauma-informed, and context-sensitive approaches to pediatric catatonia.

## Review

Aim

This structured qualitative synthesis of eight previously published pediatric catatonia case reports, supplemented by a targeted literature review, aimed to: (1) characterize the clinical presentation and treatment patterns of pediatric catatonia; (2) examine how SDOH were reported in relation to diagnosis, management, and clinical outcomes; (3) identify factors associated with delayed recognition and treatment; and (4) describe psychiatric, neurodevelopmental, and medical conditions associated with pediatric catatonia. This approach also sought to highlight contextual and structural barriers affecting access to care in pediatric populations.

Methods

Study Design

This study is a structured qualitative synthesis of published pediatric catatonia case reports combined with a targeted narrative literature review. Key elements of the Preferred Reporting Items for Systematic Reviews and Meta-Analyses (PRISMA) 2020 reporting framework were applied to enhance transparency in the literature search, study selection, and reporting process [20]. The aim was to examine the clinical presentation, treatment approaches, and SDOH reported in relation to diagnosis, management, and outcomes in pediatric catatonia.

Eligibility Criteria

The literature review included English-language case reports and case series describing catatonia in pediatric patients, particularly those with underlying psychiatric or neurodevelopmental conditions such as autism spectrum disorder (ASD), psychotic disorders, mood disorders, anxiety disorders, or trauma-related conditions. Articles published between February 2009 and July 2023 were eligible for inclusion. Studies were excluded if the full text was unavailable or if reports lacked sufficient clinical detail regarding patient presentation, catatonic features, treatment course, or clinical outcomes. Reports were considered relevant to the SDOH analysis when socioeconomic, cultural, structural, familial, or healthcare access factors influencing diagnosis or management were described. 

Search Strategy

A focused literature search was conducted between March 16 and March 24, 2024, with the most comprehensive search performed on March 21, 2024. Databases and literature resources searched included PubMed, Google Scholar, NIH, CDC, and WHO. Representative search strings included combinations of the terms "pediatric catatonia," "autism spectrum disorder," "psychosis," "trauma," "benzodiazepines," "electroconvulsive therapy," and "social determinants of health" using Boolean operators. 

Study Selection

A total of 178 records were identified through the initial literature search. Titles, abstracts, and full-text articles were screened by two authors to assess relevance to pediatric catatonia, underlying psychiatric or neurodevelopmental conditions, and the influence of SDOH. Following initial screening, 114 records were excluded due to lack of relevance. 64 full-text articles were assessed for eligibility, of which 56 were excluded because of insufficient clinical detail or limited relevance to the study aims. Eight case reports met inclusion criteria and were included in the final qualitative synthesis. The study selection process is summarized in a PRISMA flow diagram (Figure 1).

**Figure 1:**
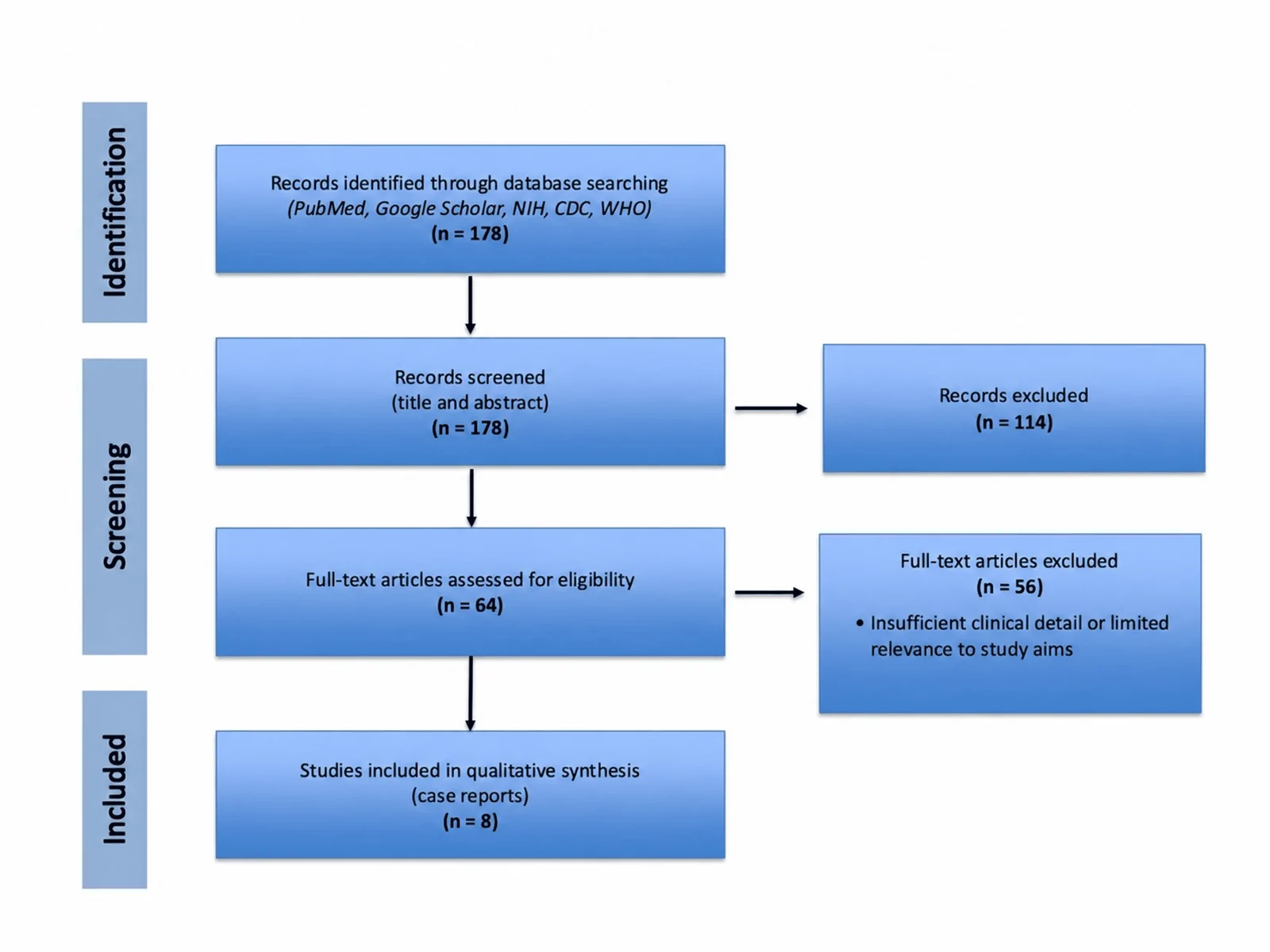
PRISMA flow diagram illustrating the study selection process for the targeted literature review A total of 178 records were identified through database searching. After title and abstract screening, 114 records were excluded. 64 full-text articles were assessed for eligibility, of which 56 were excluded due to insufficient relevance or clinical detail. Eight case reports were included in the final qualitative synthesis. The study selection process has been presented using the PRISMA 2020 framework [20]. PRISMA: Preferred Reporting Items for Systematic Reviews and Meta-Analyses

Data Extraction

Data extraction was performed manually by two authors using a standardized format to ensure consistency across included reports. Extracted variables included patient characteristics (age and sex), underlying psychiatric or neurodevelopmental diagnoses, clinical presentation and subtype of catatonia, treatment modalities (including benzodiazepines, antipsychotics, and ECT), and reported outcomes. Demographic background was defined as any racial, ethnic, national, or ancestry descriptor explicitly reported in the original case report. No demographic category was inferred from treatment location or author affiliation, and information not explicitly reported by the source authors was recorded as “Not Reported.” SDOH were also recorded when available, including access to psychiatric care, socioeconomic factors, housing stability, family dynamics, cultural considerations, and community support. Extracted data were reviewed by both authors for completeness and accuracy and were organized into a comparative table to facilitate pattern identification across cases.

Quality Assessment

The methodological quality of the included case reports was assessed using the Joanna Briggs Institute (JBI) Critical Appraisal Checklist for Case Reports [21]. The JBI checklist is a validated appraisal instrument designed for case reports and evaluates eight domains: patient demographics, clinical history and timeline, current clinical condition, diagnostic assessment, intervention or treatment, post-intervention clinical condition, adverse events or harms, and takeaway lessons. Each included case report was reviewed using this checklist to assess completeness of reporting and suitability for qualitative synthesis.

Given the descriptive and exploratory nature of this targeted narrative review, the JBI checklist was used to support transparent appraisal rather than to generate weighted quantitative estimates. Case reports were included if they provided sufficient clinical detail regarding diagnosis, catatonic features, treatment course, and outcome. Reports were also reviewed for the presence of contextual information relevant to SDOH when available. All eight case reports were appraised as suitable for inclusion in the qualitative synthesis using the JBI Critical Appraisal Checklist for Case Reports (Table 1).

**Table 1:** JBI critical appraisal of included case reports Critical appraisal of the eight included case reports using the JBI Critical Appraisal Checklist for Case Reports. Each report was evaluated across eight domains: (1) patient demographics, (2) patient history and timeline, (3) current clinical condition, (4) diagnostic assessment and methods, (5) intervention or treatment procedures, (6) post-intervention clinical condition, (7) adverse events or unanticipated events, and (8) key takeaway lessons. “Y” indicates Yes, “N” indicates No, “U” indicates Unclear, and “NA” indicates Not Applicable. Overall appraisal was recorded as Include, Exclude, or Seek Further Information in accordance with the JBI Critical Appraisal Checklist for Case Reports. All eight reports were considered suitable for inclusion in the qualitative synthesis. JBI: Joanna Briggs Institute

Case / Reference	Q1: Demographics	Q2: History / Timeline	Q3: Presentation	Q4: Diagnostic Assessment	Q5: Intervention	Q6: Post-intervention Condition	Q7: Adverse Events / Harms	Q8: Takeaway Lessons	Overall Appraisal
Case 1 / Bozkurt and Mukaddes [12]	Y	Y	Y	Y	Y	Y	Y	Y	Include
Case 2 / Quigley et al. [13]	Y	Y	Y	Y	Y	Y	N	Y	Include
Case 3 / Micaldi et al. [14]	Y	Y	Y	Y	Y	Y	Y	Y	Include
Case 4 / Ahmed et al. [15]	Y	Y	Y	Y	Y	Y	N	Y	Include
Case 5 / Ridgeway et al. [16]	Y	Y	Y	Y	Y	Y	Y	Y	Include
Case 6 / Weiss et al. [17]	Y	Y	Y	Y	Y	Y	Y	Y	Include
Case 7 / Traverso et al. [18]	Y	Y	Y	Y	Y	Y	Y	Y	Include
Case 8 / Tedesco et al. [19]	Y	Y	Y	Y	Y	Y	N	Y	Include

Data Synthesis

No quantitative synthesis or meta-analysis was performed because of the heterogeneity and descriptive nature of the included reports. Cases were reviewed for shared clinical features, underlying psychiatric or neurodevelopmental conditions, treatment strategies, and outcomes. Patterns related to response to benzodiazepines, adjunctive use of antipsychotics, and utilization of ECT were identified. Observations related to SDOH were examined in relation to diagnostic delay, treatment accessibility, and clinical outcomes. Only case reports appraised as suitable for inclusion using the JBI Critical Appraisal Checklist were included in the qualitative synthesis. Cases were subsequently compared for overlapping clinical features, underlying psychiatric or neurodevelopmental diagnoses, treatment modalities, outcomes, and SDOH.

Results

Eight pediatric patients with catatonia were included in this qualitative synthesis (Table 2). Ages ranged from 8 to 17 years (mean age 13 years; median age 12.5 years). The sample included five female and three male patients. Underlying psychiatric and neurodevelopmental diagnoses included ASD or Asperger’s disorder (Cases 1, 2, and 7), schizoaffective disorder (Case 3), major depressive disorder with anxious distress (Case 5), psychotic disorders (Cases 6 and 8), and a trauma-related disorder (Case 4).

**Table 2:** Clinical and demographic characteristics of pediatric patients with catatonia Each case corresponds to previously published case reports [12-19]. Demographic background reflects racial, ethnic, national, or ancestry descriptors as reported in the original case reports. Terminology was retained as originally reported and was not standardized or reclassified. ASD: Autism spectrum disorder; ECT: Electroconvulsive therapy

Case	Age	Sex	Demographic Background	Primary Diagnosis	Catatonia Subtype	Treatment Approach	Outcome
1 [12]	11	M	Not Reported	ASD	Stuporous	Benzodiazepines; supportive care	Improved
2 [13]	13	F	Caucasian	Asperger's Disorder	Excited	Benzodiazepines; antipsychotic	Resolved
3 [14]	16	F	African American	Schizoaffective disorder	Stuporous	Benzodiazepines; antipsychotic	Improved
4 [15]	12	F	Not Reported	Trauma-related disorder	Stuporous	Benzodiazepines; ECT	Stabilized
5 [16]	12	M	Not Reported	Major depressive disorder with anxious distress	Stuporous	Benzodiazepines	Improved
6 [17]	8	F	Marshallese	Psychotic disorder	Excited	Benzodiazepines; antipsychotic	Improved
7 [18]	15	M	Not Reported	ASD	Stuporous	Benzodiazepines; supportive care	Partial improvement
8 [19]	17	F	Mexican descent	Schizophrenia	Stuporous	Benzodiazepines; antipsychotic	Remission

Treatment Patterns

Catatonia subtypes varied across cases, with stuporous presentations most common (Cases 1, 3, 4, 5, 7, and 8), followed by excited (Cases 2 and 6). Benzodiazepines were associated with clinical improvement or stabilization across all eight cases. Antipsychotic medications were used as adjunctive therapy in cases with prominent psychotic features (Cases 2, 3, 6, and 8). ECT was used in one of eight cases (Case 4).

SDOH

SDOH and other psychosocial or structural factors were heterogeneously reported across the included case reports (Table 3). Documented factors included bereavement and family stress, foster-care involvement and trauma exposure, mental-health stigma, barriers to accessing specialized treatment, immigrant or rural context, bullying, and socioeconomic hardship. Several reports explicitly described barriers that affected recognition of symptoms or access to treatment, whereas others provided limited or no SDOH-specific information. These observations are descriptive and should not be interpreted as demonstrating a causal relationship between individual social determinants and catatonia severity or outcome. 

**Table 3:** Social and contextual factors, barriers to care, and clinical course reported in included cases SDOH: Social determinants of health; ECT: Electroconvulsive therapy; ASD: Autism spectrum disorder

Case	Source-Reported SDOH/Contextual Factors	Source-Reported Barrier to Recognition or Care	Relevant Clinical Course
1 [12]	Bereavement following death of father; school/new-environment stress	Grief-related behavioral changes were not initially recognized as such; insufficient support and treatment for grief/depressive symptoms	Progressive behavioral regression, withdrawal, and mutism preceding catatonia; marked improvement with lorazepam
2 [13]	Not clearly reported	Not clearly reported	Clinical catatonia course described, but no specific SDOH-related barrier or outcome could be verified from the available report
3 [14]	Foster-care/state-custody involvement; parental loss; history of possible physical/sexual abuse and sex trafficking	Fragmented collateral information and incomplete prior records; multiple placements, hospitalizations, and agencies involved in care	Diagnostic clarification was difficult and occurred after a prolonged, complex psychiatric course
4 [15]	Severe interpersonal trauma; mental-health stigma	Father initially delayed psychiatric evaluation because of fear of mental-health stigma	Symptoms worsened before psychiatric presentation, with severe catatonia, poor oral intake, and dehydration; ultimately treated with benzodiazepines and ECT
5 [16]	Significant family stress related to father's psychiatric illness	Pediatric ECT access barriers, including cultural barriers, High Court approval requirements, lack of an experienced pediatric ECT center, and COVID-19-related referral limitations	Progressive functional decline and catatonia; ECT was not available, while high-dose lorazepam resulted in substantial improvement
6 [17]	Immigrant Marshallese family living in a rural area	No specific patient-level healthcare-access or language barrier was explicitly reported	Prolonged treatment course requiring high-dose benzodiazepines; recurrence of catatonic symptoms occurred with benzodiazepine reduction or accidental discontinuation
7 [18]	Bullying and severe psychosocial stress	Diagnostic complexity related to overlap between ASD and catatonia; family subsequently lost confidence in medications and declined additional pharmacologic interventions	Prolonged fluctuating course with partial improvement and incomplete return to baseline functioning
8 [19]	Low socioeconomic status and financial strain; sociocultural mental-health stigma; language/cultural factors; school stressors	Family disagreement regarding psychiatric illness and treatment; stigma and access/communication barriers contributed to delayed intervention	Psychotic illness progressed for approximately one year before definitive treatment; catatonia subsequently improved with lorazepam and risperidone

Discussion

Clinical Presentation and Predisposing Conditions

This study highlights the heterogeneity of pediatric catatonia presentations, consistent with previously described stuporous, excited, malignant, and periodic subtypes [1]. Common features included mutism, rigidity, waxy flexibility, and psychomotor retardation in stuporous presentations, while excited catatonia manifested with agitation and self-injurious behavior, as observed in an eight-year-old female patient with childhood-onset psychosis [17].

Neurodevelopmental and psychiatric disorders emerged as frequent predisposing factors. ASD was present in multiple cases. In the original clinic-based study by Wing and Shah, catatonia was identified in approximately 6% of the total ASD sample and in approximately 17% of referrals aged 15 years or older [22]. More recently, a systematic review and meta-analysis estimated the pooled prevalence of catatonia in individuals with ASD at 10.4% (95% confidence interval (CI), 5.8%-18.0%) [23].

Catatonia in ASD

Recognition of catatonia in individuals with ASD is particularly challenging due to symptom overlap, including mutism, stereotypies, and agitation. This diagnostic overlap often results in delayed recognition and treatment. Catatonic symptoms in individuals with ASD commonly emerge during adolescence or early adulthood [22,23].

Response to benzodiazepines in ASD-associated catatonia has been variable, with some patients demonstrating partial or minimal improvement and others experiencing paradoxical effects or excessive sedation. In contrast, ECT is an established treatment for catatonia that is refractory to benzodiazepines and has been successfully used in pediatric populations [5,6]. Diagnostic overshadowing, where new symptoms are attributed to baseline ASD features, remains a significant barrier to timely intervention [18,24]. Trauma, deprivation, and abuse have also been described as important contextual factors in pediatric catatonia, particularly in cases without an identified medical etiology [25].

Psychosis-Associated Catatonia and Treatment Considerations

Psychotic disorders were among the most frequently observed primary diagnoses in this qualitative synthesis and in prior epidemiologic studies, accounting for a substantial proportion of pediatric catatonia cases [26]. In psychosis-associated catatonia, symptom severity was often pronounced, necessitating combined pharmacologic strategies. Benzodiazepines served as first-line treatment, while atypical antipsychotics such as risperidone and quetiapine provided adjunctive benefit in patients with prominent psychotic features. In refractory or malignant presentations, ECT emerged as a critical, life-saving intervention, though access remained limited by systemic and legislative constraints [5].

Trauma, Supportive Care, and SDOH

Supportive care, including hydration, nutritional support, and physical stabilization, was essential in preventing secondary complications such as dehydration and malnutrition, emphasizing the need for multidisciplinary management.

Socioeconomic stressors, healthcare access barriers, cultural stigma, and trauma were reported in relation to diagnostic delay, treatment access, and clinical course in several cases. In some cases, mental health stigma and other sociocultural barriers contributed to delayed help-seeking and access to psychiatric treatment [15,19]. Trauma, deprivation, and abuse have been described as important contextual factors in pediatric catatonia, particularly in cases without an identified medical etiology [25]. 

Limitations

This study has several limitations that should be considered when interpreting the findings. The qualitative synthesis includes a small number of published cases, which limits generalizability and precludes statistical analysis. The accompanying literature review was targeted and qualitative in nature rather than a formal systematic review. Although study selection and data extraction were independently reviewed by two authors, the targeted review design may still introduce the potential for selection bias. Additionally, the analysis relied on information reported in published case reports, which varied in the depth and completeness of clinical and social determinant data. As a qualitative synthesis of published case reports, causal relationships between SDOH and clinical outcomes cannot be established. Despite these limitations, this study highlights important clinical patterns and contextual factors relevant to the recognition and management of pediatric catatonia.

## Conclusions

This study underscores the diagnostic and therapeutic complexity of pediatric catatonia, highlighting the wide spectrum of clinical presentations and the importance of considering SDOH and other contextual factors in pediatric catatonia. Common predisposing factors included neurodevelopmental disorders, such as ASD, and psychiatric conditions, particularly psychotic disorders. Early recognition and intervention remain critical, as untreated catatonia can result in severe morbidity or mortality. Across cases, benzodiazepines, particularly lorazepam, demonstrated consistent efficacy as first-line treatment. Atypical antipsychotics, including risperidone and quetiapine, provided additional benefit in cases with psychotic features. In refractory presentations, ECT proved to be a life-saving intervention, though access remains constrained by systemic and legislative barriers. Supportive care measures were essential in managing severe catatonic states.

Delays in diagnosis and treatment were frequently associated with socioeconomic hardship, family instability, trauma exposure, and cultural stigma. Addressing these systemic barriers through increased clinical awareness, trauma-informed care, and equitable access to evidence-based treatment is essential to improving outcomes for pediatric patients with catatonia.

## References

[1] Vargas JG, da Costa e Silva BF, Butler BJ, Meyerovitz C, Waldman L (2022). Catatonia successfully treated with lorazepam and clozapine in an adolescent with first-episode psychosis: a case report. Psychiatry Res Case Rep.

[2] Edinoff AN, Kaufman SE, Hollier JW (2021). Catatonia: Clinical overview of the diagnosis, treatment, and clinical challenges. Neurol Int.

[3] von Känel S, Nadesalingam N, Alexaki D, Baumann Gama D, Kyrou A, Lefebvre S, Walther S (2022). Measuring catatonia motor behavior with objective instrumentation. Front Psychiatry.

[4] Bush G, Fink M, Petrides G, Dowling F, Francis A (1996). Catatonia. I. Rating scale and standardized examination. Acta Psychiatr Scand.

[5] Dhossche DM, Wachtel LE (2010). Catatonia is hidden in plain sight among different pediatric disorders: a review article. Pediatr Neurol.

[6] Sienaert P, Dhossche DM, Vancampfort D, De Hert M, Gazdag G (2014). A clinical review of the treatment of catatonia. Front Psychiatry.

[7] Thakur A, Jagadheesan K, Dutta S, Sinha VK (2003). Incidence of catatonia in children and adolescents in a paediatric psychiatric clinic. Aust N Z J Psychiatry.

[8] Ghaziuddin N, Hendriks M, Patel P, Wachtel LE, Dhossche DM (2017). Neuroleptic malignant syndrome/malignant catatonia in child psychiatry: literature review and a case series. J Child Adolesc Psychopharmacol.

[9] (2026). Social Determinants of Health. https://odphp.health.gov/healthypeople/priority-areas/social-determinants-health.

[10] Dhossche DM, Withane N (2019). Electroconvulsive therapy for catatonia in children and adolescents. Child Adolesc Psychiatr Clin N Am.

[11] Grenier E, Ryan M, Ko E, Fajardo K, John V (2011). Risperidone and lorazepam concomitant use in clonazepam refractory catatonia: a case report. J Nerv Ment Dis.

[12] Bozkurt H, Mukaddes NM (2010). Catatonia in a child with autistic disorder. Turk J Pediatr.

[13] Quigley J, Lommel KM, Coffey B (2009). Catatonia in an adolescent with Asperger's disorder. J Child Adolesc Psychopharmacol.

[14] Micaldi SC, Dao CW, Napier DN, Samson J (2021). A case of pediatric catatonia. Kans J Med.

[15] Ahmed GK, Elbeh K, Karim AA, Khedr EM (2021). Case report: catatonia associated with post-traumatic stress disorder. Front Psychiatry.

[16] Ridgeway L, Okoye A, McClelland I, Dhossche D, Kutay D, Loureiro M (2021). Case report: a case of pediatric Catatonia: role of the lorazepam challenge test. Front Psychiatry.

[17] Weiss MD, Schibuk L, Gokarakonda SB, Henderson R, Esmaeilpour D (2022). Catatonia with psychosis in an 8-year-old child: a case report and a literature review. Case Rep Psychiatry.

[18] Traverso A, Ancora C, Zanato S, Raffagnato A, Gatta M (2021). Diagnostic and therapeutic challenges of catatonia in an adolescent with high functioning autism spectrum disorder: a case report. Front Psychiatry.

[19] Tedesco S, Gonzalez R, Owusu-Antwi P, Robinson RE, Janusz C (2023). A case of undiagnosed schizophrenia with catatonia in a Hispanic adolescent: the significance of social determinants in the diagnosis and the efficacy of risperidone and lorazepam in treatment. Cureus.

[20] Page MJ, McKenzie JE, Bossuyt PM (2021). The PRISMA 2020 statement: an updated guideline for reporting systematic reviews. Int J Surg.

[21] Moola S, Munn Z, Tufanaru C (2020). Chapter 7: systematic reviews of etiology and risk. JBI Manual for Evidence Synthesis.

[22] Wing L, Shah A (2000). Catatonia in autistic spectrum disorders. Br J Psychiatry.

[23] Vaquerizo-Serrano J, Salazar De Pablo G, Singh J, Santosh P (2021). Catatonia in autism spectrum disorders: a systematic review and meta-analysis. Eur Psychiatry.

[24] Moore S, Amatya DN, Chu MM, Besterman AD (2022). Catatonia in autism and other neurodevelopmental disabilities: a state-of-the-art review. Npj Ment Health Res.

[25] Dhossche DM, Ross CA, Stoppelbein L (2012). The role of deprivation, abuse, and trauma in pediatric catatonia without a clear medical cause. Acta Psychiatr Scand.

[26] Luccarelli J, Kalinich M, Fernandez-Robles C, Fricchione G, Beach SR (2022). The incidence of catatonia diagnosis among pediatric patients discharged from general hospitals in the United States: a kids’ inpatient database study. Front Psychiatry.

